# Study–test congruence of response levels in item stimulus–response priming

**DOI:** 10.3758/s13421-020-01021-9

**Published:** 2020-02-21

**Authors:** Carlos A. Gomes, Andrew Mayes

**Affiliations:** 1 Memory Research Unit, Division of Neuroscience and Experimental Psychology, University of Manchester, Manchester, M13 9PL UK; 2 Department of Neuropsychology, Ruhr-University Bochum, 44801 Bochum, Germany

**Keywords:** Implicit memory, Repetition priming, Novel associations, Stimulus–response learning, Instances

## Abstract

We investigated stimulus–response (S–R) memory links during object priming using a binary associative size judgement paradigm. At study, participants decided which of two objects was bigger in real life and, at test, made the same or the reverse judgement. We examined the effects of response congruence on item S–R priming in the associative paradigm. In Experiment 1, a task reversal manipulation had minimal impact on RT priming when classifications were congruent for both recombined objects between study and test. Experiment 2 found that RT priming was more disrupted by classification incongruence of the selected than of the nonselected item alone, with incongruence of the nonselected object having no effect on RTs. Experiment 3, however, found that classification incongruence of both items eliminated RT priming, indicating that a significant effect of classification incongruence for the nonselected item is only evident if both items are classification-incongruent. Finally, across all experiments, we found that accuracy was more sensitive than RTs to decision/action incongruence. We interpret these findings in light of a two-stream account of S–R priming, and suggest a few extensions to account for interactions between S–R links of recombined items.

**Electronic supplementary material:**

The online version of this article (10.3758/s13421-020-01021-9) contains supplementary material, which is available to authorized users.

Priming is a kind of implicit memory in which the processing of a stimulus during a learning phase leads to more accurate or faster response times (RTs) to that stimulus when the same response is made during a subsequent test phase (Richardson-Klavehn & Bjork, 1988; Roediger, 1990; Tulving & Schacter, 1990). Although such priming is found even when conscious memory of the stimulus is absent (Gomes, Mecklinger, & Zimmer, 2019; Gomes, Montaldi, & Mayes, 2015; Hamann & Squire, 1997), it may sometimes support successful recognition memory (Gomes, Mecklinger, & Zimmer, 2017; Taylor & Henson, 2012; Voss, Lucas, & Paller, 2012).

Research suggests that priming during binary classification tasks involves forming a direct memory link between a stimulus representation and its task-based response representation(s), the direct retrieval of which, in an appropriate test situation, speeds responding or makes it more accurate (Denkinger & Koutstaal, 2009; Dobbins, Schnyer, Verfaellie, & Schacter, 2004; Horner & Henson, 2009; Rothermund, Wentura, & De Houwer, 2005; Waszak, Hommel, & Allport, 2004). Several theories about the underlying mechanisms of this kind of stimulus–response (S–R) priming have been proposed, such as Logan’s instance theory (Logan, 1990, 1997) or Hommel’s event file theory (Hommel, 1998, 2004). These theories assume that a stimulus and its task-based response are encoded together in episodic memory in an event file or instance. When the stimulus is repeated, the stored event file or instance containing the response is automatically retrieved. This often has a facilitatory effect, since it effectively allows bypassing much of the processing engaged during the initial exposure to the stimulus.

In a series of elegant studies, Horner and Henson (Horner & Henson, 2009, 2011, 2012) have helped to develop an even more sophisticated view of response processing and representation that plays a key role in item S–R priming^1^ (see also Denkinger & Koutstaal, 2009; Giesen & Rothermund, 2016; Moutsopoulou, Yang, Desantis, & Waszak, 2015). They used binary classification tasks, in which single object stimuli are presented at study and test and participants are asked to judge whether each stimulus is, for example, bigger or smaller than a reference object, such as a shoebox. Horner and Henson have provided evidence that single-item S–R priming depends on three levels of response: action, decision, and classification. At the top level, the classification response takes the form “the chair is bigger than a shoebox” whether the question asked is the same (“Is the chair bigger than the shoebox?”) or the reverse (“Is the chair smaller than the shoebox?”). At the next level down, the classification response can be indicated by more than one kind of decision response, depending on the precise form of the question asked (e.g., yes/no or selecting chair/selecting shoebox could indicate the same classification response). At the lowest level, the same decision response could be indicated by different action responses (e.g., pressing either left or right keys, or making different vocalizations could all indicate the same decision response choice).

Horner and Henson selectively varied whether each level of response at test is congruent (matches) or incongruent (mismatches) with the response at study, by changing the test task (e.g., “Is the chair bigger than a shoebox?” at study to “Is the chair smaller than a shoebox?” at test) or changing the reference object (e.g., “shoebox” at study to “wheelie bin” at test). By using congruence manipulations like these, it was shown that incongruence at some or all response levels reduced RT item S–R priming (Allenmark, Moutsopoulou, & Waszak, 2015; Denkinger & Koutstaal, 2009; Gomes & Mayes, 2015a; Horner & Henson, 2009, 2011, 2012; Moutsopoulou, Pfeuffer, Kiesel, Yang, & Waszak, 2018; Moutsopoulou & Waszak, 2012; Pfeuffer, Hosp, et al., 2018a; Pfeuffer, Moutsopoulou, Waszak, & Kiesel, 2018b). Horner and Henson further posited that priming is determined by the interaction between the retrieval of congruent/incongruent S–R bindings and the recomputation of the appropriate response. This “two-stream” account thus implies that incongruence has the potential to reduce or even eliminate priming because incorrect responses are automatically triggered and interfere with the efficient generation of correct responses.

There have been far fewer studies of S–R priming with novel associations between items. In a typical associative priming paradigm, participants are presented with preexperimentally unrelated item pairs at study. At test, some of these pairs are repeated (intact condition), some pairs consist of a recombination of items that appeared in different pairs at study (recombined condition), and some are novel pairs (new condition). A significant performance advantage in the intact relative to the recombined condition indicates associative priming, whereas a similar advantage for the recombined relative to the novel pair condition indicates item priming (Gomes & Mayes, 2015b; Goshen-Gottstein, Moscovitch, & Melo, 2000; Kan et al., 2011). Thus, in the associative paradigm, responses can be bound to the association between the two object items (indicating associative priming) as well as to each item of a pair (indicating single-item priming).

There are several reasons why research on item S–R priming, as measured in associative tasks, is important. First, current S–R theories of repetition priming often extrapolate the ideas from single-item S–R priming research to associative S–R priming. However, as considered below, item S–R priming measured using the associative paradigm may have distinct properties from its single-item counterpart. Thus, a comprehensive S–R theory may have difficulty explaining both item priming for object pairs and single-item priming. Second, in daily life, we seldom encounter items in isolation, but rather in combination with other items or contexts, so the associative S–R priming paradigm is ecologically relevant. In addition, because the retrieval of S–R links of paired items may occur outside awareness (Gomes & Mayes, 2015b), it may automatically influence how we consciously relate those items, making the study of associative priming even more pertinent. Third, investigating the circumstances under which response incongruence bias responding occurs could have important implications in decision-making in general.

As few studies have used binary classification associative tasks, it is unclear whether the effects of incongruence on item S–R priming in associative tasks relate to incongruence effects for single-item priming. A full answer to this question needs to acknowledge a distinctive feature of the associative priming paradigm. There are two items that are paired in each trial, one is selected at test, whereas the other is nonselected at test. For example, if the pair was “train–elephant” and the test question was “Which is bigger?”, the selected item would be “train”, and the nonselected item would be “elephant”. This relationship may not be reciprocal; isolated incongruence of selected versus nonselected items may have different effects on item S–R priming.

Dennis and Schmidt (2003) conducted a study in which study word pairs such as “desk–jeep” and “tea bag–flowerpot” would be recombined into “desk–flowerpot” at test; the task “Which is the bigger object?” was used at both study and test. In this case, there was incongruence at all three levels of response for both the selected item (i.e., “desk”) and the nonselected item (i.e., “flowerpot”; e.g., study-to-test classification for “desk” changed from “smaller” to “bigger” and decision/action changed from “do not select desk” to “select desk”).^2^ Dennis and Schmidt (2003) found that incongruent recombined pairs were responded to more slowly and less accurately than congruent recombined pairs.

In another associative priming study, Dew and Giovanello (2010b) found no reduction in RT item S–R priming when the size judgement task was reversed from study to test. In their study, item pairs were constructed such that reversing the task did not change object-level classification responses between study and test phases. For example, if “desk–squirrel” and “violin–flowerpot” were the studied item pairs, and “desk–flowerpot” the recombined test pair, both “desk” and “flowerpot” would retain their classifications (i.e., desk and flowerpot would be the bigger and smaller items, respectively, at both study and test). However, reversing the task (from “Which is bigger?” at study to “Which is smaller?” at test) did lead to decision/action response incongruence for both the selected item (e.g., desk was the chosen item at study, but should not be selected at test) and nonselected item (e.g., flowerpot was not the chosen item at study, but had to be selected at test). Because decision/action responses were congruent for selected and nonselected items in the “same” task, one would have expected less item S–R priming in the recombination condition in the “reverse” than “same” task, if, as suggested by the single-item priming literature, S–R incongruence at the decision/action level affects both accuracy and RT item priming (Denkinger & Koutstaal, 2009; Dennis & Perfect, 2013; Horner & Henson, 2009; Pfeuffer, Hosp, et al., 2018a; Pfeuffer, Pfister, Moutsopoulou, Waszak, & Kiesel, 2017).

The present study had two major aims. First, we examined whether item S–R priming in the associative paradigm is sensitive to the same manipulations that affect it in the single-item paradigm. As we did find that item S–R priming in the associative paradigm had discordant properties from those related to single-item S–R priming, our second aim was to shed light on what mechanism could be driving association-based item S–R priming. To achieve this, we manipulated study-to-test response incongruence of item pairs in a hierarchical fashion. Specifically, in Experiment 1, neither the selected nor the nonselected item had incongruent classifications. In Experiment 2, classification incongruence occurred for either the selected or nonselected item alone. In Experiment 3, both the selected and nonselected items were classification incongruent. Decision/action incongruence also varied linearly across the three experiments (although in opposing directions for “same” and “reverse” tasks; see Table 1). By comparing incongruence effects at decision/action and classification response levels on item S–R priming in this paradigm, we aimed to advance understanding of interactive competition between processes underlying different response levels.

**Table 1:**
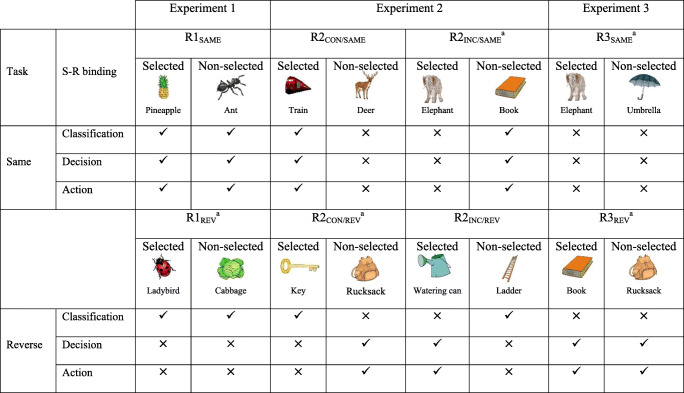
Congruence of classification, decision, and action stimulus–response (S–R) bindings for recombined pairs for each test task in Experiments 1–3

Note. R1 = recombined pairs in which both the selected and nonselected objects had congruent classifications; R2_CON_ = recombined pairs in which the selected object had a congruent classification and the nonselected object an incongruent classification at test; R2_INC_ = recombined pairs in which the selected object had an incongruent classification and the nonselected object a congruent classification at test; R3 = recombined pairs in which both the selected and nonselected objects had an incongruent classification at test; Selected = object which was the correct response at test and, therefore, should be selected; Nonselected = object which was not the correct response at test, and, therefore, should not be selected. The ticks (✓) and crosses (×) refer to whether the corresponding S–R binding is congruent or incongruent, respectively, during the test task.

^a^ The left–right positioning of the objects in pairs R1_REV_, R2_INC/SAME_, R2_CON/REV_, R3_SAME_, and R3_REV_ is reversed in this table (see Figs. 1, 3, and 5 for the correct positioning) because we wished to make the selected/nonselected columns consistent in the table, which facilitates comparisons across pair types

## Experiment 1

In Experiment 1, participants performed an associative size-judgement task at study (“Which is bigger?”) and, at test, made the same or the reverse judgement (“Which is smaller?”). This experiment let us ascertain whether reversing the task at test, which caused the decision/action (but not classification) responses to become incongruent (see Table 1), disrupted RTs or accuracy. We also manipulated amount of study (one vs. three study presentations). Research shows that single-item priming is greater after stimuli are presented multiple times at study (Horner & Henson, 2009; Moutsopoulou et al., 2015; Pfeuffer, Moutsopoulou, et al., 2018). It could be that the insensitivity of item priming to a task reversal in Dew and Giovanello’s study was the result of weakly encoded S–R links.

### Method

#### Participants

Forty-eight undergraduate students took part in our study in exchange for monetary compensation or course credits (age range: 18–33 years). A power analysis (effect size based on Gomes & Mayes, 2015b) revealed that detection of a medium effect of item priming with a power of .80 would require a minimum size of 22 participants. All participants in this and subsequent experiments gave written consent before the beginning of the experiment, and all had normal or corrected-to-normal vision.

#### Materials

The materials used and the construction of object pairs followed previous research in this area (Dew & Giovanello, 2010a, 2010b; Gomes, Figueiredo, & Mayes, 2016; Gomes & Mayes, 2015b). Two hundred and fifty-two (240 for the experiment and 12 for practice) coloured clip-art images of common objects were selected from an Internet clip-art database (www.clipart.com). These pictures were scaled down to fit in a box of 400 × 400 pixels, so as not to create a response bias for larger images. The objects were carefully selected to have an unambiguous size in real life and similar line complexity. The 240 study–test pictures were split into 30 groups, each containing eight pictures. The pictures in each group were further divided into two subgroups of four pictures with the restriction that the pictures in a subgroup were unrelated to the pictures in the other subgroup. Two different word association norms (Moss & Older, 1996; Nelson, McEvoy, & Schreiber, 2004) were used to ensure the absence of any preexisting relationship between the objects. This was achieved by selecting pairs that, first, did not belong to the same semantic category (e.g., two pictures of animals were never paired together) and, second, were not produced together in the word association norms mentioned above. Three independent native English judges cross-checked whether the objects in each subgroup were unambiguously bigger than the objects in the other subgroup.

Once the selection and validation procedures were completed, the four pictures within a subgroup were randomly paired with the four pictures in the other subgroup, giving a total of 120 item pairs (Dew & Giovanello, 2010a, 2010b; Gomes & Mayes, 2015b). There were 40 trials per condition at test (intact, recombined, and new), half of which were presented in the “same” and the other half in the “reverse” task. For any given condition in any given task, an equal number of right-sided and left-sided objects were the “bigger” objects. The position of the pictures on the screen remained constant between study and test phases.

#### Design and data analysis

The design comprised test task (same, reverse) and pair type (recombined, new) as within-subject factors, and task order (same/reverse, reverse/same) and prime level (low, high) as between-subject factors. This paper focused on item priming as measured in a novel associative task, so we omitted intact pairs from all analyses (the results from the intact condition will be presented elsewhere). In addition, task order did not systematically interact with the other factors, so, to simplify statistical analyses, we do not report it further.

In order to ensure that comparisons of conditions across tasks were not biased due to, for example, the “reverse” task being more difficult to execute, which might increase RTs or errors, we computed baseline-corrected scores (for raw mean values for this and subsequent experiments, see Supplementary Table S1 in the Supporting Information). For error data, we subtracted error rates for recombined pairs from new pairs (i.e., new − recombined) for each test task separately (accuracy priming measure). For RT data, we computed proportional item priming (new – recombined / new) also for each test task separately (proportional priming measure; see Supporting Information for the results of the analyses using subtractive RTs). Both our accuracy and proportional RT priming measures have been previously validated and are standard in S–R priming studies (Denkinger & Koutstaal, 2009; Horner & Henson, 2009; Schnyer, Dobbins, Nicholls, Schacter, & Verfaellie, 2006). Only item pairs with correct responses at test were included in the RT analysis. Item priming scores were then submitted independently to a 2 (prime level: one, three) × 2 (test task: same, reverse) mixed repeated-measures ANOVA. A Huynh–Feldt correction was applied to the degrees of freedom of those tests for which the assumption of sphericity was violated. Planned comparisons were conducted to investigate item priming separately for each test task and prime level. The alpha level was set, for all statistical tests, at .05 and were two-tailed, unless stated otherwise. Effect sizes are reported in the form of Cohen’s *d* and partial eta-squared (η^2^_p_) where appropriate.

#### Procedure

**Fig. 1:**
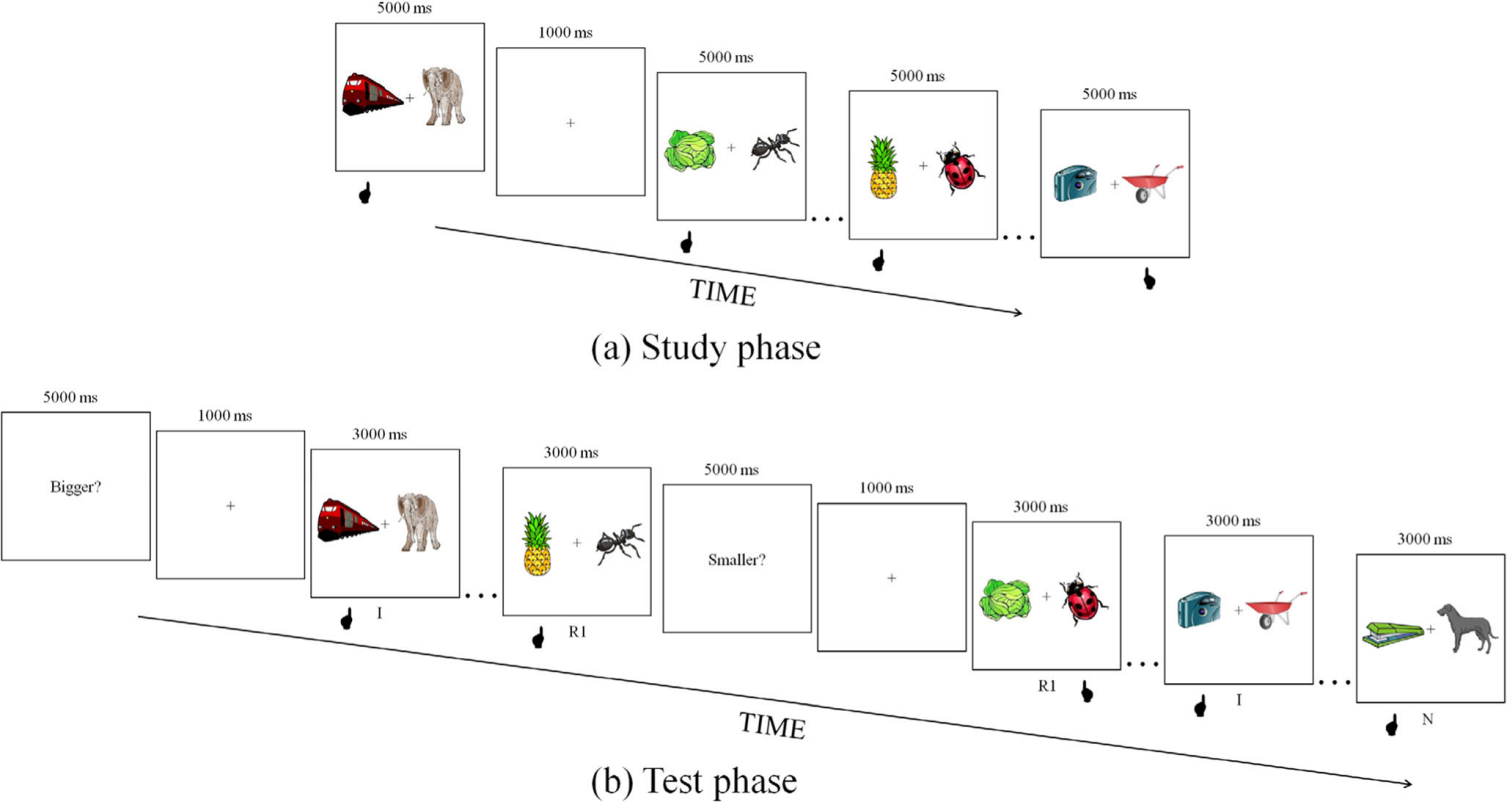
Experimental design of Experiment 1. At study (**a**), participants decided which object of a pair was bigger in real life. At test (**b**), they performed the “same” task (Bigger?) or the “reverse” task (Smaller?). Hand under each event points to the selected (correct) item. The number above each event corresponds to the duration of that event in milliseconds. I = intact pairs; R1 = recombined pairs; N = new pairs. *Note.* In the text and in Table 1, we also refer to R1 pairs shown in the “same” and “reverse” task as R1_SAME_ and R1_REV_, respectively

Figure 1 shows the experimental design used in the current experiment. At study, participants saw pairs of object pictures and were instructed to decide which object was bigger in real life. They used the left–right control keys on a standard computer keyboard to decide whether the left–right object was bigger, respectively. A fixation cross was displayed for 1,000 ms at the beginning of each trial followed by the pair of pictures for up to 5,000 ms. Twenty-four participants saw each pair of pictures once (low primed condition), whereas the other 24 saw each pair three times in three separate runs (high primed condition; each run with a new random presentation order of the same pairs). At test, participants were told that they would need to perform two different tasks: in the “same” task, they were required to decide which object in the pair was bigger in real life, whereas in the “reverse” task, they were asked to judge which object was smaller in real life. The order of the tasks was counterbalanced across participants. They were asked to try to respond as quickly and accurately as possible. The first trial started with the presentation of a cue word (either Bigger? or Smaller?) for 5,000 ms, indicating which of the two tasks participants were about to perform (the other cue was shown half-way through the experiment), followed by a fixation cross for 1,000 ms. A picture pair, comprising one of the three possible types of association (intact, recombined or new), was subsequently presented and remained on the screen for 3,000 ms, within which time participants responded. The MATLAB (http://www.mathworks.com) toolbox Cogent (http://www.vislab.ucl.ac.uk/cogent.php) was used to present stimuli and record participants’ responses.

### Results

In order to obtain a representative index of S–R binding, and to conform to other studies of this kind (e.g., Horner & Henson, 2009), errors, coded as either an incorrect response or an absence of a response during the priming, as well as outlying trials with RTs that were more than two standard deviations (*SD*s) above or below the mean value of each condition, were removed from subsequent analyses.^3^ This procedure resulted in the elimination of approximately 10% of the total amount of trials for all participants and conditions.

**Fig. 2:**
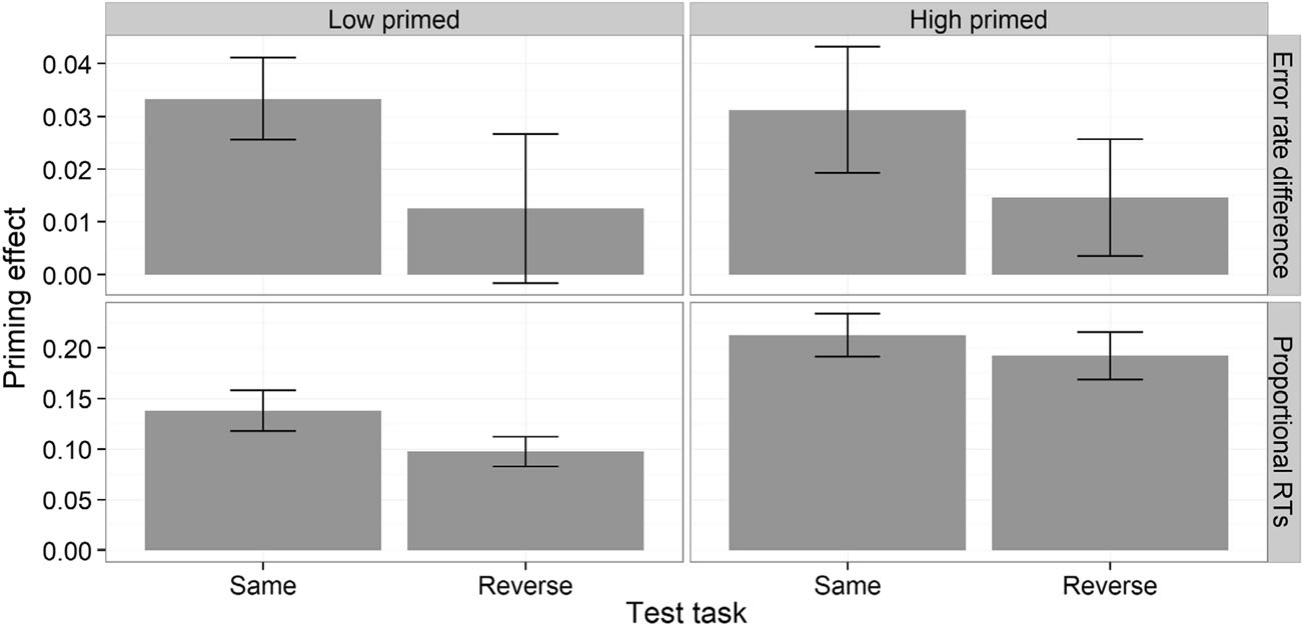
Difference in error rates (new − recombined; top panel) and proportional RT priming (new – recombined / new; bottom panel) for the recombined condition during the “same” and “reverse” tasks split by prime level (low, high) in Experiment 1. Error bars represent the standard error of the mean. *Note.* In the text and in Table 1, we also refer to R1 pairs shown in the “same” and “reverse” task as R1_SAME_ and R1_REV_, respectively

Difference error rate scores (accuracy priming) were computed by subtracting recombined from new pairs (see Fig. 2, top). A 2 (prime level) × 2 (test task) mixed repeated-measures ANOVA on these difference scores did not reveal either a significant main effect of test task, *F*(1, 46) = 2.30, *p* > .10, η^2^_p_ = .05, or an interaction, *F*(1, 46) = .03, *p* > .10, η^2^_p_ = .001. Despite the absence of a main effect of test task, we decided to test single effects, given that previous studies have reported significant differences between “same” and “reverse” tasks. Accuracy priming was indeed significant in the “same” task in both prime level conditions (both *t*s > 2.61, *p*s < .05, *d*s > 0.53), whereas it was nonsignificant in the “reverse” task (both *t*s < 1.32, ps > .10).

Next, proportional RT item priming scores were computed to account for the different baseline RTs within each test task (see Fig. 2, bottom). There was robust proportional item priming in both prime level conditions and test tasks (all *t*s > 6.57, *p*s < .001). Despite a numerical advantage for “same” (.17) versus “reverse” (.14) item priming, a 2 (prime level) × 2 (test task) mixed repeated-measures ANOVA, revealed a main effect only of prime level, *F*(1, 46) = 15.80, *p* < .001, η^2^_p_ = .26, which, unsurprisingly, indicated greater priming for the high than low primed condition. Neither the main effect of test task, *F*(1, 46) = 2.64, *p* > .10, η^2^_p_ = .05, nor the interaction, *F*(1, 46) = .28, *p* > .10, η^2^_p_ = .01, were significant, suggesting that RT item priming in this experiment was not affected by a task reversal. Indeed, the difference between “same” and “reverse” priming was not significant when tested separately for each prime level condition (*t*s < 1.80, *p*s > .09).

The above interpretation, however, was based on a null finding, so, we decided to conduct a Bayes factor (BF) analysis to determine the strength of evidence favouring the alternative hypothesis of a genuine difference between “same” and “reverse” tasks. A BF value smaller than 1/3 or greater than 3 is commonly interpreted as substantial evidence, whereas anything in between is only anecdotal evidence. The BF for the comparison between the alternative (difference between “same” and “reverse”) and the null (no difference between the tasks) hypothesis was 0.67, suggesting that, even though there is not much evidence to distinguish between the hypotheses, the data do not preferentially favour the alternative hypothesis.

### Discussion

Contrary to what has been found with the single-item paradigm, RT item priming, as measured during an associative paradigm, was not significantly reduced in the “reverse” relative to the “same” task. Although this conclusion rests on a null result and should, therefore, be interpreted with caution, it is consistent with the results of a very similar study (Dew & Giovanello, 2010b). Our BF analysis also did not provide substantial evidence for the alternative hypothesis (BF = 0.67). Finally, we have conducted the same experiment (although in an MRI scanner) using a different sample and again could not find differences in RT priming between “same” and “reverse” tasks.

One possibility why RT item priming in the present experiment may have been insensitive to a task reversal could be because this kind of priming resulted purely from the reinstatement of perceptual and/or conceptual information from the study phase. Alternatively, RTs could have been more sensitive to classification incongruence than decision/action incongruence. Given that classifications were congruent for both recombined items in the “same” and “reverse” tasks (see Table 1), the lack of a significant effect of “same” versus “reverse” item priming would be unsurprising.

When accuracy was used as a measure, however, item priming appeared to be present in the “same” (e.g., *d* = .87 for the low-primed condition) but not in the “reverse” task (e.g., *d* = .14 for the low-primed condition). This finding indicates that the influence of incongruent decision/action levels of response on recombinations may be affecting accuracy to a greater extent than it is influencing RTs. We should note, however, that the main effect of test task was nonsignificant, so caution must be exercised interpreting these simple effects. Experiment 2 sheds additional light on decision/action levels of response incongruence, so this issue will be considered more thoroughly in the General Discussion.

Finally, multiple study trials did not increase the cross-task priming effect, as there was no Prime Load × Test Task interaction. One criticism could be that given that prime level was manipulated across participants, those in the three-presentation condition required more time to perform the experiment than participants in the one-presentation condition. Although we find it unlikely that this influenced the results, it is, nevertheless, a potential confound we did not eliminate.

## Experiment 2

Experiment 1 revealed that item S–R priming, as measured during associative tasks, may involve a different mechanism compared with single-item S–R priming. Experiment 2 was conducted to try and determine what this mechanism may be. In the previous experiment, item-level classification responses for recombined objects were congruent between study and test phases. This arrangement helped to ensure that any difference between recombined and new pairs was not the result of a change in the relative classification status of recombined objects. Evidence that altering the classification status of associations between study and test affects the magnitude of item S–R priming in associative tasks comes from two studies from Dennis and colleagues (Dennis, Carder, & Perfect, 2010; Dennis & Schmidt, 2003). These authors found that classification-congruent recombined pairs were judged faster and more accurately than classification-incongruent recombined pairs.

A critical aspect of Dennis and colleagues’ studies is that both the selected and nonselected items in each recombined pair were either congruent at test with what they had been at study or both were incongruent (see Table 2). To the extent that S–R bindings act on each component of an association independently (Giesen, Frings, & Rothermund, 2012; Giesen & Rothermund, 2014, 2016), it is possible that response incongruence for the selected and nonselected objects affects priming differently in the recombined condition. However, because Dennis and colleagues did not manipulate classification incongruence for selected and nonselected objects separately, whether incongruence classification effects are larger when the selected rather than the nonselected object suffers a classification change is unclear.

**Table 2:** Congruence of classification, decision, and action stimulus–response (S–R) bindings for recombined pairs in Dennis and Schmidt’s (2003) and Dennis et al.’s (2010) studies

Test task	S–R binding	Recombined congruent		Recombined incongruent	
		Selected	Nonselected	Selected	Nonselected
Same	Classification	✓	✓	×	×
	Decision	✓	✓	×	×
	Action	✓	✓	×	×
		Recombined congruent		Recombined incongruent	
		Selected	Nonselected	Selected	Nonselected
Reversea	Classification	✓	✓	×	×
	Decision	×	×	✓	✓
	Action	×	×	✓	✓

*Note.* Selected = object which was the correct response at test and, therefore, should be selected; Nonselected = object which was not the correct response at test, and, therefore, should not be selected. The ticks (✓) and crosses (×) refer to whether the corresponding S–R binding is congruent or incongruent, respectively, during the test task.

^a^The “reverse” condition was only included in the Dennis et al.’s (2010) study

Experiment 2 sought to understand the independent effects of response incongruence on the selected and nonselected objects in the recombined condition. This may help disentangle the relative contribution of each item’s S–R bindings to the item priming effect observed in Experiment 1. To achieve this goal, we manipulated the recombination condition incongruence separately for the selected and nonselected items.

### Method

#### Participants

Forty-eight undergraduate students were recruited in exchange for monetary compensation or course credit (age range: 19–30 years).

#### Materials

Two-hundred and sixty-eight (256 for the experiment and 12 for practice) coloured clip-art images were selected and went through the same selection procedures and relatedness checks as those described in the Method section of Experiment 1. For the present experiment, two lists containing a total of 64 pairs (128 pictures) were formed. Within each list, eight groups were created. Next, we will describe how pairs were created within List 1 (the creation of pairs within List 2 was identical but, for counterbalancing purposes, the relative size of the objects was reversed in each subgroup). Each group consisted of four subgroups (A, B, C, D), with four pictures of objects each. The relative size of the objects in each group followed the pattern A > B = C > D, meaning that A objects were the biggest, D objects the smallest, and B and C objects had an identical size (for List 2 the pattern was B > A = C > D). The pictures in Subgroups A and D were always presented on the left side of the screen, whereas pictures in Subgroups B and C were presented on the right side. Study pairs were created by randomly pairing the pictures of Subgroups A and D to the pictures of Subgroups B and C, respectively. A total of 96 item pairs were presented at study.

**Fig. 3:**
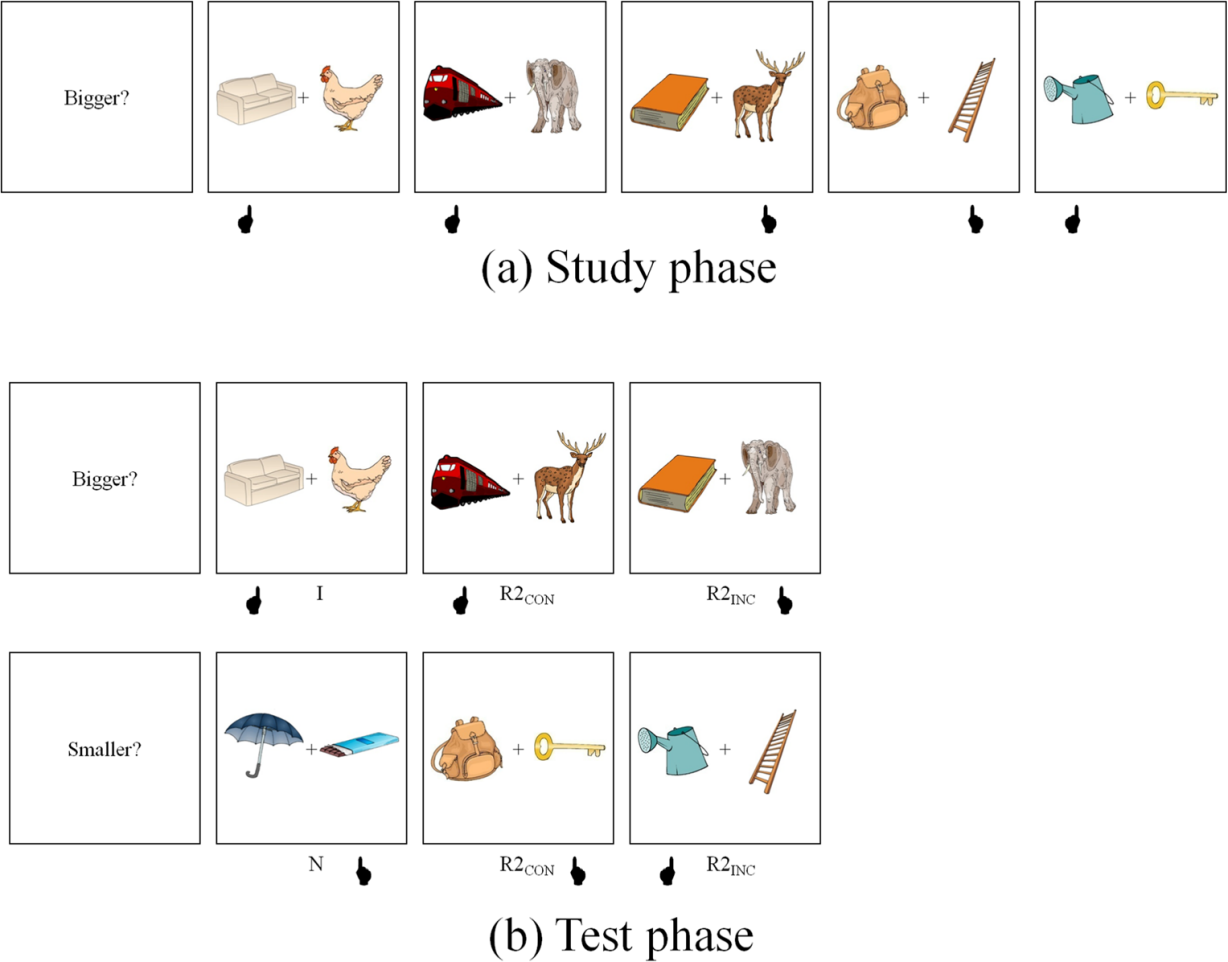
Experimental design of Experiment 2. At study (**a**), participants decided which object of a pair was bigger in real life. At test (**b**), they performed the “same” task (Bigger?) or the “reverse” task (Smaller?). Hand under each event points to the correct answer. I = intact pairs; R2_CON_ = recombined pairs in which the selected object had a congruent classification and the nonselected object an incongruent classification at test; R2_INC_ = recombined pairs in which the selected object had an incongruent classification and the nonselected object a congruent classification at test; N = new pairs. *Note.* In the text and in Table 1, we also refer to R2_CON_ and R2_INC_ pairs shown in the “same” task as R2_CON/SAME_ and R2_INC/SAME_, respectively. Similarly, we refer to R2_CON_ and R2_INC_ pairs shown in the “reverse” task as R2_CON/REV_ and R2_INC/REV_, respectively

To construct the two types of recombined pairs, for each group, the pictures in Subgroups A and D were randomly recombined with the pictures of Subgroups C and B, respectively. Thus, the objects in Subgroups A and D (e.g., “train” and “book” in Fig. 3) maintained their relative classification status between study and test phases, whereas the objects in Subgroups B and C (e.g., “elephant” and “deer”) changed classification between experimental phases. Critically, for A–C recombinations, the selected object at test maintained its relative classification status (i.e., R2_CON_^4^ in Fig. 6). In contrast, for D–B recombinations, the selected object at test had an incongruent classification, whereas the nonselected object had a congruent classification (i.e., R2_INC_ in Fig. 3). Importantly, R2_CON_ and R2_INC_ were matched in terms of object-level response congruence as well as size-judgement difficulty (see Supporting Information). A total of 128 pairs (32 intact, 32 R2_CON_, 32 R2_INC_, 32 new) were presented in the test phase.

#### Design and procedure

We computed difference scores between each recombined type and new pairs (e.g., R2_CON_ − new), as well as proportional RT scores for each recombined type (e.g., [new− R2_CON_] / new). Thus, the experimental design for this experiment consisted of congruence (R2_CON_, R2_INC_) and test task (same, reverse) as within-subject factors and prime level (low, high) as a between-subjects factor. The statistical analysis performed on these data followed a similar pattern to that of Experiment 1, and the procedure was identical (see Fig. 3).

### Results

Approximately 9% of trials were excluded for all participants and conditions using the exclusion criteria described in Experiment 1.

**Fig. 4:**
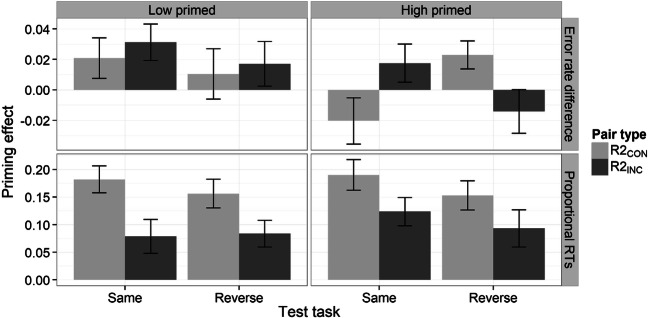
Difference in error rates (new − recombined; top panel) and proportional RT priming (new – recombined / new; bottom panel) for R2_CON_ and R2_INC_ pairs during the “same” and “reverse” tasks split by prime level (low, high) in Experiment 2. Error bars represent the standard error of the mean. *Note.* In the text and in Table 1, we also refer to R2_CON_ and R2_INC_ pairs shown in the “same” task as R2_CON/SAME_ and R2_INC/SAME_, respectively. Similarly, we refer to R2_CON_ and R2_INC_ pairs shown in the “reverse” task as R2_CON/REV_ and R2_INC/REV_, respectively

Figure 4 shows the difference in error rates (top) and proportional RTs (bottom), for each congruence type (R2_CON_, R2_INC_) split by test task (same and reverse) and prime level (low, high).

Difference in accuracy scores (accuracy priming) were submitted to a 2 (prime level: low, high) × 2 (congruence: R2_CON_, R2_INC_) × 2 (test task: same, reverse) repeated-measures ANOVA. There was a significant Congruence × Test Task interaction, *F*(1, 46) = 7.80, *p* < .01, η^2^_p_ = .15, as the result of greater accuracy priming for R2_CON/REV_ (.02) relative to R2_CON/SAME_ (.001), but greater accuracy priming for R2_INC/SAME_ (.02) relative to R2_INC/REV_ (.002). The three-way interaction also reached significance, *F*(1, 46) = 6.68, *p* < .05, η^2^_p_ = .13, which suggested that the differences above were present for the high primed condition but not for the low primed condition (see Fig. 4, top).

Regarding RT data, a 2 (prime level) × 2 (congruence) × 2 (test task) repeated-measures ANOVA revealed a significant main effect only of congruence, *F*(1, 46) = 58.31, *p* < .001, η^2^_p_ = .56, indicating that recombinations with classification congruence for the selected object alone (R2_CON_) were judged faster than recombinations with classification incongruence for the selected object alone (R2_INC_) regardless of test task (R2_CON_ = .17 vs. R2_INC_ = .09; see Fig. 4, bottom). Nevertheless, item priming was significant for both R2_CON_ and R2_INC_ in both test tasks (all *t*s > 4.3, *p*s < .001). Note that the main effect of classification congruence was properly matched for both test task and decision/action congruence—R2_CON_ and R2_INC_ differed only in that the classification was congruent for the selected object and incongruent for the nonselected object for R2_CON_, whereas it was the other way round for R2_INC_ (see Table 1).

Even though the interaction between congruence and test task was not significant, we decided to directly perform two separate contrasts (collapsed across prime level): (1) R2_CON/SAME_ versus R2_INC/REV_ RT priming, and (2) R2_CON/REV_ versus R2_INC/SAME_ RT priming. These contrasts can be thought of as pure tests of the interaction between classification congruence and object selection. This is because, for R2_CON/SAME_ (and R2_CON/REV_), the classification was congruent for the selected object, but incongruent for the nonselected object, whereas for R2_INC/REV_ (and R2_INC/SAME_), it was the other way round. Critically, responses at the decision/action levels between the two types of recombination were matched in both Contrast 1 and 2 (see Table 1). As predicted, in both contrasts, participants were slower when the classification incongruence occurred for selected item, relative to when the classification incongruence occurred for the nonselected item, Contrast 1 (R2_CON/SAME_ vs. R2_INC/REV_), *t*(47) = 4.24, *p* < .001, *d* = .63; Contrast 2 (R2_CON/REV_ vs. R2_INC/SAME_), *t*(47) = 2.21, *p* < .05, *d* = .29. This result not only indicates that the congruence effect was due to classification-specific incongruence, but also suggests that the congruence effect was not a consequence of reinstating the same task at test, because, in Contrast 2, “reverse” task recombinations showed speedup relative to “same” task recombinations.

Given that item priming declined more when classification incongruence was for the selected object, we wondered how important response congruence for the nonselected object was in our associative task. For that purpose, we compared R2_CON/SAME_ with R1_SAME_ because these two types of recombination only differed for the nonselected object (compare these two conditions in Table 1). There was no difference in proportional RT item priming between the two recombinations, *t*(94) = .48, *p* > .10, *d* = .10, which suggests that classification incongruence of nonselected objects on their own does not disrupt item RT priming.

### Discussion

The results of the present experiment indicated that classification incongruence of the selected item has a much bigger effect on RT S–R priming than incongruence of the nonselected object. This was indicated by faster RTs for R2_CON_ (congruent classification for the selected item and incongruent classification for the nonselected), than R2_INC_ (incongruent classification for the selected item and congruent classification for the nonselected item). Nevertheless, RTs for both kinds of recombination were faster than those for new pairs in both test tasks, revealing some item priming.

The difference in performance between R2_CON_ and R2_INC_ cannot be simply explained by changes in *item-level* classification status: Both R2_CON_ and R2_INC_ contained one item with a congruent classification (e.g., “train” for R2_CON/SAME_ and “ladder” for R2_INC/REV_; see Table 1) as well as one item with an incongruent classification (e.g., “deer” for R2_CON/SAME_ and “watering can” for R2_INC/REV_; see Table 1). Likewise, object-level decision/action responses were also matched across these two recombination types, so this result cannot be due to differences in decision/action bindings.

Another important finding was the observation that incongruence for the nonselected object had a negligible impact on the RT item priming effect, since R1_SAME_ (which had congruent S–R bindings for both selected and nonselected items) did not show additional RT priming than R2_CON/SAME_ (which had congruent S–R bindings for the selected item but incongruent S–R bindings for the nonselected item). This result could explain why RT priming was similar between R1_SAME_ and R1_REV_ in Experiment 1, since both conditions had congruent classifications for the selected item.

Regarding accuracy, we observed reduced accuracy priming when decision/action incongruence occurred for the nonselected object (e.g., R2_CON/SAME_) relative to when it occurred for the selected object (e.g., R2_CON/REV_), although this effect emerged only in the high primed condition. Some studies have failed to observed S–R priming effects when only stimulus–action bindings changed between study and test (Hsu & Waszak, 2012; Schnyer et al., 2007), which has led to the suggestion that action bindings may be relatively weak (Hsu & Waszak, 2012; Moutsopoulou et al., 2015; Pfeuffer, Hosp, et al., 2018a; Pfeuffer, Moutsopoulou, et al., 2018b). Thus, it is possible that multiple study trials may be necessary to strengthen stimulus–action bindings to a level that can be detected in these kinds of classification experiments.

## Experiment 3

In the previous experiment, we did not find a difference in the magnitude of RT priming between recombinations in which all levels of response representation for both the selected and nonselected object were congruent (R1) relative to recombinations in which all levels of response representation for the nonselected object alone were incongruent (R2_CON/SAME_). From this finding, we concluded that S–R bindings of the nonselected item may have played a minor role in item S–R priming detected in Experiment 1. However, an alternative possibility could be that the effect of incongruence on nonselected objects only becomes detectable when the classification status of the selected object is also incongruent. If so, the retrieval of S–R bindings for the nonselected item may only operate when the utility of S–R bindings for the selected object becomes compromised. Experiment 3 was designed to test this hypothesis by changing congruence of both selected and nonselected items at test.

### Method

#### Participants, materials, procedure, and design

Forty-six undergraduate students were recruited in exchange for monetary compensation or course credits (age range: 19–35 years). The materials were largely taken from Experiment 2, and the formation of associations followed a similar procedure with the following exceptions. Within each list, eight groups were created. For List 1, each group consisted of four subgroups (A, B, C, D), each with four pictures of objects. The relative size of the objects in each subgroup followed the pattern A > B > C > D, meaning that the objects in Subgroup A were the biggest, followed by the objects in Subgroup B, followed by the objects in Subgroup C, and finally by the objects in Subgroup D (for List 2 the pattern was D < C < B < A). The pictures in Subgroups A and C were always presented on the left side of the screen, whereas the pictures in Subgroups B and D were always presented on the right side of the screen. Study pairs were created by randomly pairing the pictures of Subgroups A and C to the pictures of Subgroups B and D, respectively. Four groups were randomly selected for the intact condition, eight groups for the recombined condition (four for each of the two recombination types; see below) and four groups for the new condition.

**Fig. 5:**
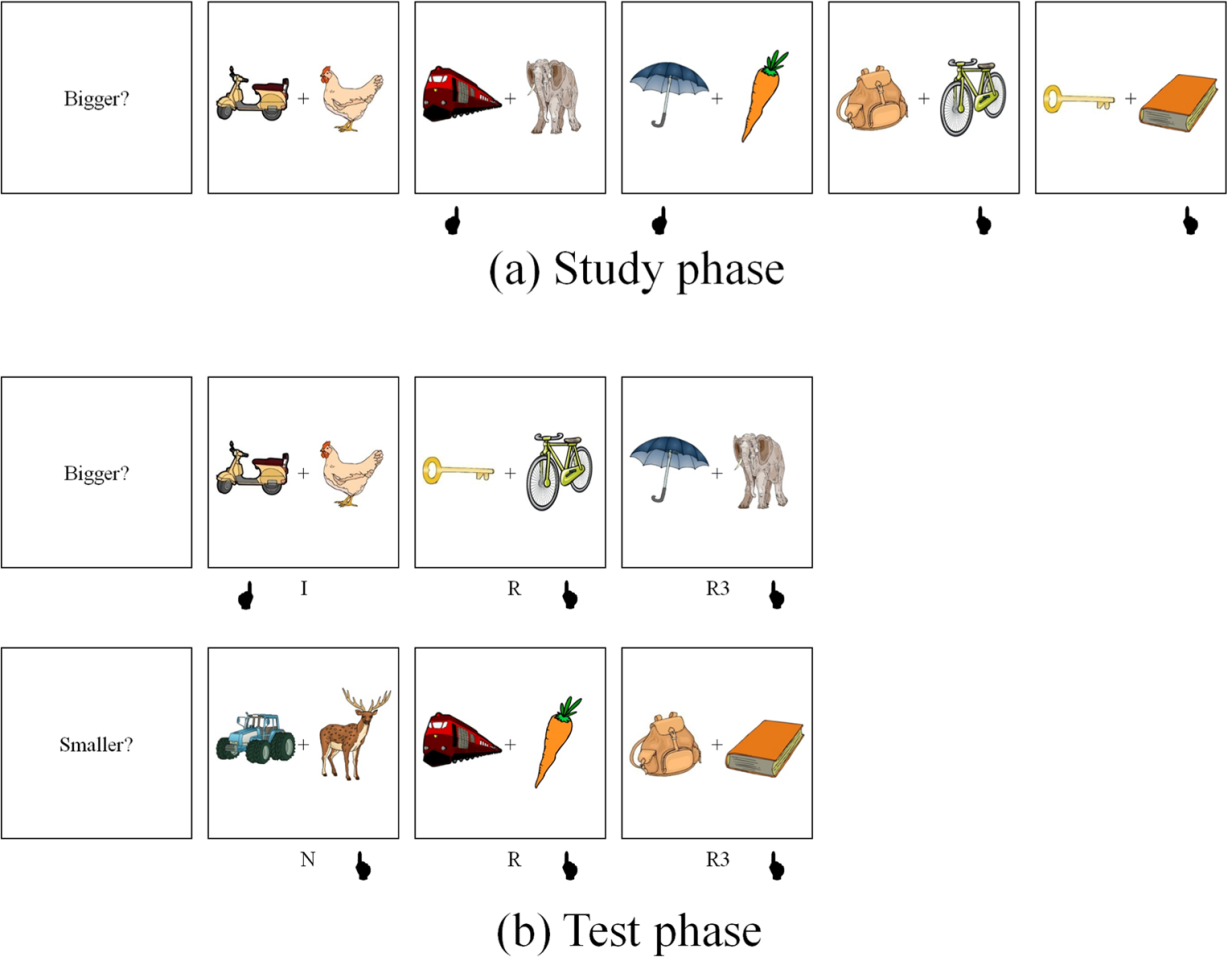
Experimental design of Experiment 3. At study (**a**), participants decided which object of a pair was bigger in real life. At test (**b**), they performed the “same” task (Bigger?) or the “reverse” task (Smaller?). Hand under each event points to the selected (correct) item. I = intact pairs; R3 = recombined pairs in which both the selected and nonselected objects had an incongruent classification at test; R = recombined pairs in which neither object suffered a classification change at test (these pairs were not included in the analyses; see text); N = new pairs. *Note.* In the text and in Table 1, we also refer to R3 pairs shown in the “same” and “reverse” tasks as R3_SAME_ and R3_REV_, respectively

To construct the two types of recombined pairs, for each group, the pictures in Subgroups A and C were randomly recombined with the pictures of Subgroups D and B, respectively. Thus, both objects in A–D associations (R, e.g., “train–carrot”, in Fig. 5) maintained their relative classification status between study and test phases, whereas both objects in B–C associations (R3, e.g., “umbrella–elephant”, in Fig. 5) suffered a change in classification status between experimental phases. Half of the R and R3 were assigned to the “same” task, whereas the other half was assigned to the “reverse” task. Importantly, R3 and new pairs were matched in terms of size-judgement difficulty; R pairs were not included in the analysis because these pairs were necessarily easier to classify (due to the selection procedure described above) than R3 or new pairs (see Supporting Information).

The procedure was identical to that of the previous experiment and is exemplified in Fig. 5. The design consisted of test task (same, reverse) as within-subject factors and prime level (high, low) as a between-subject factor.

### Results

**Fig. 6:**
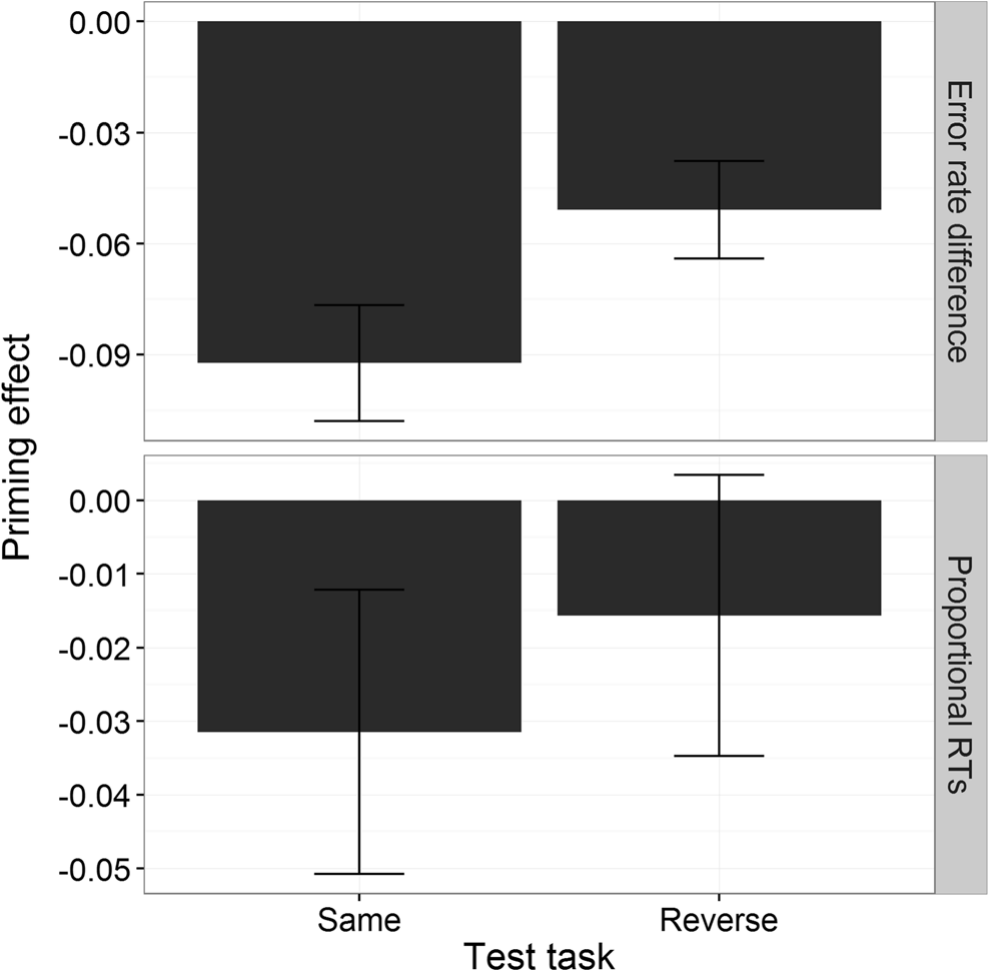
Difference in error rates (new − recombined; top panel) and RT proportional priming (new – recombined / new; bottom panel) during the “same” and “reverse” tasks in Experiment 3. Error bars represent the standard error of the mean. *Note****.*** In the text and in Table 1, we also refer to R3 pairs shown in the “same” and “reverse” tasks as R3_SAME_ and R3_REV_, respectively

Approximately 12% of trials were excluded for all participants and conditions using the exclusion criteria described in Experiment 1. Figure 6 shows the error rate difference (accuracy priming; top) and proportional RT priming (bottom) for R3 split by test task (same and reverse).

A 2 (prime level) × 2 (test task) mixed repeated-measures ANOVA on accuracy priming only yielded a significant main effect of test task, *F*(1, 46) = 4.60, *p* < .05, η^2^_p_ = .09, with lower accuracy priming for R3 in the “same” (−.09) than in the “reverse” (−.05) task (all other *F*s < 1.7, *p*s > .10). Collapsed across prime level, a negative accuracy priming effect was observed for both R3_SAME_, *t*(47) = −5.86, *p* < .001, *d* = .81, and R3_REV_, *t*(47) = −3.87, *p* < .001, *d* = .56.

For the proportional RT item priming analysis, a 2 (prime level) × 2 (test task) mixed repeated-measures ANOVA did not reveal any significant main effects or interaction (all *F*s < 2.28, *p*s > .10). In stark contrast to the previous two experiments, recombined pairs appeared to have been judged slower than baseline pairs during both test tasks (see Fig. 6, bottom). Item priming was not significant for the “reverse” task, *t*(47) = −.80, *p* > .10, *d* = .11, and there was even a modest negative priming for the “same” task, *t*(47) = −1.64, *p* = .05, *d* = .24 (one-tailed). BF analysis revealed that, in both tasks, the null hypothesis of no priming was favoured relative to the alternative hypothesis of positive priming (BFs < 0.15).

To test the importance of nonselected item incongruence, we compared the RT priming effect between R2_INC/SAME_ and R3_SAME_ (these differ only at the level of the nonselected object; see Table 1). The difference was highly significant, *t*(94) = 4.77, *p* < .001, *d* = .96, indicating greater proportional RT priming for R2_INC/SAME_ than R3_SAME_. One could argue that the lack of priming in the present experiment resulted from the fact that the present items were more difficult to prime than those in Experiment 2. However, baseline RTs were identical in both experiments, *t*(94) = 1.05, *p* > .29, *d* = .22, so it is highly unlikely that this result could be explained by this kind of difficulty confound. Finally, we also replicated the outcome congruence effect reported by Dennis and colleagues (see Supporting Information).

### Discussion

In the present experiment, we observed greater RT priming for recombinations in which only the selected object had incongruent responses (R2) relative to when both the selected and nonselected objects had incongruent responses (R3). This result provides strong evidence that the retrieval of S–R links associated with the nonselected object does contribute to the general item priming effect.

Interestingly, our results showed that R3 did not significantly differ from new item pairs in either test task, indicating that item RT priming in the present experiment was not obtained. In fact, there was some evidence of negative RT priming occurring for the “same” task, as well as negative accuracy priming in both tasks (see Fig. 6). These results differ from those of Dennis and colleagues, in that they observed *facilitation* for completely incongruent recombined pairs relative to new pairs.

There are several possible reasons for this discrepancy. First, the present experiment used pairs of pictures of objects, whereas Dennis and colleagues (Dennis & Schmidt, 2003; Dennis et al., 2010) used word pairs; thus, facilitation at the phonological level may have contributed to the residual item priming effect in their experiments, as phonological representations are more likely to be recruited during word processing relative to object processing (Damian & Bowers, 2003). Second, Dennis and colleagues gave feedback each time participants made a wrong decision, whereas no feedback was given in any of our experiments. It is possible that this feedback may have led participants to become more conservative when classifying unfamiliar (new) relative to familiar (studied) words, thus, increasing RTs and decreasing error rates for new pairs. Third, more problematic and most significant, Dennis and colleagues’ recombined-incongruent items were separated by two size steps, whereas items in new pairs were separated by only one. This means that recombined-incongruent pairs in Dennis and colleagues’ experiments would have been easier to judge than new pairs, given that the size difference between the objects in new pairs was smaller (and the decision more difficult to reach) than in recombined-incongruent pairs. In contrast, for the present experiment, we constructed R3 so that they were not easier (or more difficult) to judge than new pairs (i.e., the size difference between the objects in R3 was equivalent to that of new pairs).

## General discussion

Item S–R priming was investigated in three experiments using a novel associative priming paradigm. Experiment 1 examined whether reversing the test task, which resulted in selectively incongruent decision/action responses, disrupted accuracy or RT priming, as is typically observed in single-item priming studies. Robust RT S–R priming was observed for both test tasks, and no difference was found between “same” and “reverse” priming, even after multiple study trials. Experiment 2 showed that RT priming was significantly reduced for pairs in which the test-selected object alone had an incongruent classification, whereas, when the nonselected object alone was classification-incongruent, priming was unaffected. In contrast, accuracy priming was lower for pairs in which the nonselected object alone had incongruent decision/action responses, relative to pairs in which the selected object alone was decision/action-incongruent. In Experiment 3, we showed that item RT priming was completely abolished when classifications of both selected and nonselected items were incongruent, which indicated that, under these conditions, classification incongruence for the nonselected object did have a disruptive effect. Finally, there was evidence for *negative* accuracy priming (i.e., more errors for recombinations than new pairs), with worse performance for pairs with decision/action incongruence of both selected and nonselected items.

At first glance, our results appear to challenge current theories of S–R priming. First, the lack of sensitivity of RT priming in our associative priming paradigm after a task reversal (Experiment 1) differs from what has been found using single-item S–R priming paradigms. Also, current theories of S–R priming must explain why RT priming is disrupted when classification incongruence occurs for the selected item alone, intact when classification incongruence occurs for the nonselected item alone (Experiment 2), and absent, or even reversed, when both items are classification-incongruent (Experiment 3). Finally, accuracy seemed more dependent on decision/action than classification congruence, a result which is also not predicted by S–R learning theories.

### Horner and Henson’s two-stream account

We propose that the manner in which participants select responses about the recombined pairs of items at test in the associative priming paradigm involves the two interactive processing streams postulated by Horner, Henson, and colleagues (Henson, Eckstein, Waszak, Frings, & Horner, 2014; Horner & Henson, 2009, 2012). According to this account, the first stream is top-down, and it involves a controlled, relatively effortful and slow series of processes that are needed to ensure accurate responses. Participants process in this “algorithmic” way because they realize that some paired items have not been seen together before so their study-phase responses could now be inappropriate. Indeed, this stream should make selections no more accurately or faster for recombined pairs than for new item pairs, unless each recombined picture has been processed slightly more efficiently in relevant perceptual/semantic ways because of its exposure at study (Blaxton, 1989; Weldon, Roediger, Beitel, & Johnston, 1995).

This account proposes that priming results from the slow stream interacting positively/negatively, with another stream so as to change item priming levels. This second stream is triggered by memory of three levels of responses (classification, decision and action) made to each recombined stimulus in the previous study episodes. It involves relatively automatic and fast activation of the three levels of response that were made at study to each re-paired object picture. It works in a nonhierarchical way, so each level of response may be directly activated in about the same time, rapidly and automatically. With response incongruence, there is likely to be competition/interference with the slower effortful stream at each affected level of response. In contrast, with congruence, the slower effortful stream may receive some facilitatory energisation at each affected level of response (Henson et al., 2014).

Incongruence should be able to reduce, eliminate, or even reverse both accuracy and RT item priming, as has been shown with single-item priming tasks (Allenmark et al., 2015; Denkinger & Koutstaal, 2009; Giesen & Rothermund, 2014; Horner, 2016; Horner & Henson, 2009, 2011; Moutsopoulou & Waszak, 2012; Moutsopoulou et al., 2015; Race, Shanker, & Wagner, 2009). However, the associative priming paradigm that we used, unlike the single-item priming paradigm, allows incongruence at the decision/action/classification response levels to affect the size and direction of activation in the faster stream either for selected or nonselected items at test.

Because our study is, to the best of our knowledge, the first to explicitly look at associative effects in terms of multiple levels of response congruence, in the next sections we will describe how our results might be explained using this two-stream account.

### Decision/action congruence: Accuracy priming effects

The two-stream priming account must explain the accuracy item priming effects by indicating (1) why the fast stream disrupted accuracy more for incongruent decision/action responses for both the selected and nonselected items (R3_SAME_) than for congruent decision/action responses for both the selected and nonselected items (R3_REV_), and (2) why the fast stream disrupted accuracy more for incongruent decision/action responses for the nonselected item only (R2_CON/SAME_ and R2_INC/REV_) than incongruent decision/action responses for the selected item only (R2_CON/REV_ and R2_INC/SAME_).

Regarding the first point, the less accurate conditions had incongruent decision/action responses for both the selected and nonselected items. This means that the fast stream should have incorrectly inhibited the response for the selected item, but also incorrectly activated the response for the nonselected item. For example, for R3_SAME_ “umbrella–elephant” (see Fig. 5), “umbrella” (the nonselected item at test) is linked to an incorrect “select umbrella” response (activation), because it had been the chosen object at study. In contrast, “elephant” (the selected item at test) is linked to an incorrect “do not select elephant” response (inhibition), because it had not been the chosen object at study. This explains why R3_SAME_ (which had *incongruent* decision/action responses for both selected and nonselected items) showed worse accuracy relative to R3_REV_— R3_REV_ had *congruent* decision/action study–test responses for both the selected (appropriate activation) and nonselected (appropriate inhibition) test items (poorer accuracy for R1_REV_ than for R1_SAME_ can be explained using a similar idea, although we should note that the main effect of test task was not significant in Experiment 1).

Regarding the second point, for recombinations with incongruent decision/action responses for the nonselected item alone (R2_CON/SAME_ and R2_INC/REV_), the fast stream should have correctly activated the item that should have been selected, but also incorrectly activated the item that should not have been selected (e.g., for the R2_CON/SAME_ “train–deer”, both items are linked to a “select” response). Contrastingly, for congruent decision/action responses for the nonselected item alone (R2_CON/REV_ and R2_INC/SAME_), the fast stream should have incorrectly inhibited the item that should have been selected but also correctly inhibited the item that should not have been selected (e.g., for the R2_INC/SAME_ “book–elephant”, both items are linked to a “do not select” response). If inhibition and activation effects of the fast stream have equivalent biasing effects on response selection, the effects should cancel out in each condition, and there should be no difference between the conditions. This obviously did not happen as recombinations with “activation-incongruent” decision/action responses (e.g., R2_CON/SAME_) were less accurate than recombinations with “inhibition-incongruent” decision/action responses (e.g., R2_CON/REV_; at least in the high primed condition). One explanation could be that inappropriate fast-stream *activation* of the nonselected item response (e.g., as with R2_CON/SAME_) is very effective, whereas fast-stream *inhibition* of the selected item response (e.g., as with R2_CON/REV_) is much less effective. This makes intuitive sense: Inhibition of an item will never bias the fast stream to choose that item (since it is associated with a “do not select” response), whereas activation of an item may bias the fast stream to choose that item (since it is associated with a “select” response).

The above hypothesis indicates that the fast stream should sometimes cause rapid and wrong selection of the nonselected items that the slow stream would (appropriately) not have selected. It should, therefore, predict faster inaccurate responses for recombinations with inappropriate decision/action response activation for the nonselected item (R2_CON/SAME_ and R2_INC/REV_) than for recombinations with appropriate decision/action response inhibition for the nonselected item (R2_CON/REV_ and R2_INC/SAME_). There was indeed such a tendency (see “Analysis of incorrect response RTs - Experiments 2 and 3” in the Supporting Information).

In Experiment 3, we observed that pairs with congruent decision/action responses for both selected and nonselected items (R3_REV_) were less accurate than new pairs. The two-stream account, however, would predict no difference in error rates between these two conditions, since fast and slow streams would be in accord. Given that decision/action congruence for both items in R3_REV_ was accompanied by classification incongruence, our finding suggests that decision/action congruence must have somehow interacted with classification incongruence, although the exact mechanism underlying this interaction is unclear.

Finally, we should note that because accuracy priming effects appeared meaningful (effect sizes were medium to large), we decided to interpret these results. Nevertheless, we acknoweledge that the small magnitude of the accuracy priming effects in Experiments 1 and 2 may raise issues of reliability (in Experiment 3 there was a sufficient number of errors for a reliable analysis of error rates). It is, therefore, essential that future research uses manipulations that produce a large amount of errors in order to confirm our accuracy priming findings.

### Classification congruence: RT priming effects

The paradigm used in Experiment 2 was original in that it permitted us to determine the effects of classification incongruence on the selected and nonselected recombined objects at test individually. Specifically, we found that recombinations with congruent classification for the selected object, but incongruent for the nonselected object (R2_CON_), showed more priming than recombinations with incongruent classification for the selected object, but congruent for the nonselected object (R2_INC_; see Fig. 4, bottom). Thus, classification incongruence of the selected item alone slowed RTs more than classification incongruence of the nonselected item alone.

Two pieces of evidence suggest that the longer RTs were not partially or totally due to the reversal of the test task. First, we used proportional priming measures, which effectively take into account the baseline levels of each test task. Second, and more importantly, recombined pairs in the “reverse” task that had congruent classification for the selected item alone (R2_CON/REV_) showed more proportional RT priming than recombined pairs in the “same” task that had incongruent classification for the selected item alone (R2_INC/SAME_), which means that the pairs in the “reverse” task showed a speed-up in relation to the pairs in the “same” task.

The faster correct RTs for recombinations with congruent classification for the selected item alone (i.e., R2_CON_) suggests that the fast stream can activate the correct classification response of the selected item (e.g., “train”), whereas inappropriate activation of the wrong classification response of the nonselected item (e.g., “deer”) does not seem to occur. In contrast, slower RTs for recombinations with incongruent classification for the selected item alone (R2_INC_) suggests that the fast stream can only activate the incorrect classification response of the selected item (e.g., “elephant”), whereas activation of the correct classification for the nonselected item (e.g., “book”) does not seem to occur. This seems consistent with the finding that the effect of incongruence for the nonselected item alone was negligible—RT priming was equivalent between recombinations with incongruent classification for the nonselected item only (R2_CON/SAME_), and recombinations with congruent classifications for both selected and nonselected objects (R1_SAME_). This suggests that incongruence for the nonselected object alone played at most a minimal role in RT priming, and that the RT difference between recombinations with congruent classification only for the selected item (R2_CON_) and recombinations with congruent classification only for the nonselected item (R2_INC_) resulted exclusively from incongruence of the selected item.

However, in Experiment 3, we showed that when *both* the selected and nonselected items had an incongruent classification (i.e., R3), item priming was eliminated, suggesting that nonselected classification incongruence has an additive effect with selected classification incongruence. One explanation for this result could be that there was some form of interaction between the fast and slow streams. For example, when presented with “train–deer”, fast-stream retrieval of the classification “bigger” for the “train” would match the response generated by the slow stream (which should also classify the “train” as “bigger”). This should produce RT facilitation even if “deer” has an incongruent classification, because the system has sufficient information to generate an adequate response (i.e., if both fast and slow streams classify “train” as bigger, then “deer” must be smaller). Presumably, the classification of the associate item might even be ignored (producing no RT costs), which may explain why RT priming did not differ between recombinations with incongruent classification only for the nonselected item (R2_CON/SAME_), and recombinations with congruent classifications for both selected and nonselected objects (R1_SAME_). However, when both items have incongruent classifications (as in Experiment 3), the slow and fast stream can never be in accord, and, therefore, more time is required to inhibit previous classification bindings and recompute an appropriate classification response. This results in no priming, or even negative priming if recomputation is particularly intensive. Although speculative, the idea of the fast and slow stream interacting is at the heart of Horner and Henson’s two-stream model, and our finding appears consistent with this idea.

### Possible practical relevance of the results for important real-life situations

The associative priming paradigm used here involves situations in which participants have to make repeated binary decisions about which of two objects has more of a given property that varies along a continuum. These decisions are made about objects that can reoccur in different combinations so that the comparative judgement that is made about them may need to change. Our results indicate that certain changes in the direction of the comparative judgements that need to be made about an item across occasions leads to an increase in judgement error rates or RTs. Decisions in certain real-life situations have to be made under time pressure, and errors may have catastrophic effects even if they are rare. This might apply, for example, to certain gambling situations that involve fast decision-making on repeated events, or to certain political decisions where previous negative comparisons do not now apply. More generally, inappropriate activation or inhibition of the fast stream may lead to reasoning errors when there is considerable time pressure to decide. There are similarities to the idea of fast and slow thinking (Kahneman, 2011). If errors are sufficiently disastrous, developing and applying the two-stream account may be valuable for guarding against them.

The raw data will be made available on researchgate. Other materials for any of the experiments will be made available upon request.

#### Author note

This work was supported by the Fundação para a Ciência e a Tecnologia under Grant SFRH/BD/41637/2007 and the University of Manchester. We thank Catherine Dibble and Katherine Jones for their assistance with data collection.

## Electronic supplementary material

ESM 1 (DOCX 63 kb)

## Footnotes

1 In this article, we use the term “S-R priming” to refer to priming obtained in binary classification tasks (e.g., Which is bigger?; Is it animate/inanimate?). The work of Horner, Henson and colleagues strongly suggests that priming during binary tasks can be explained mostly, if not solely, by S-R learning. Thus, given that all our experiments reported here use a classification binary task, we assumed that any effects we obtained resulted mostly from the acquisition and retrieval of S-R bindings.

2 Note that, as with Dennis and Schmidt’s experiment, our paradigm also did not allow us to disentangle stimulus–decision from stimulus–action bindings. A change in decision (e.g., “select right item” to “select left item”) necessarily entailed a change in action (e.g., “press right key” to “press left key”). A manipulation such as flipping the left–right position of the items from study to test phases would allow action and decision responses to be manipulated orthogonally. We did not do this, however, because we wished to keep the same perceptual configuration of the recombinations between study and test phases.

3 We also analyzed the data (1) by applying different standard deviation cutoffs (e.g., 3 *SD*), (2) without excluding outliers, and (3) using the median instead of the mean. The results were identical in all of these cases. However, because the majority of studies in this field use a cutoff of two standard deviations above and below the mean, we opted for this cutoff number.

4 The subscript in R2 pairs (e.g., _CON_) always refers to the classification congruence of the selected object. The nonselected object had always the opposite classification congruence of the selected object (see Table 1 and the main text for more details).
