# Supplementary material for: Study–test congruence of response levels in item stimulus–response priming
Source: Mem Cognit. 2020 Feb 21;48(5):839–55. doi: 10.3758/s13421-020-01021-9 (PMC7320069; doi:10.3758/s13421-020-01021-9)
Supplement: Supplementary file 1 — (DOCX 63 kb) [file 13421_2020_1021_MOESM1_ESM.docx]

**Supporting information**

| Experiment | Condition | Error rates | | | | RTs | | | |
| --- | --- | --- | --- | --- | --- | --- | --- | --- | --- |
|  |  | Low primed | | High primed | | Low primed | | High primed | |
|  |  | Same | Reverse | Same | Reverse | Same | Reverse | Same | Reverse |
| Experiment 1 |  |  |  |  |  |  |  |  |  |
|  | R1 | .03 (.01) | .04 (.01) | .03 (.01) | .04 (.01) | 897 (29) | 973 (39) | 911 (43) | 926 (32) |
|  | New | .07 (.01) | .05 (.01) | .06 (.01) | .05 (.01) | 1,054 (45) | 1,080 (42) | 1,170 (57) | 1,166 (51) |
| Experiment 2 |  |  |  |  |  |  |  |  |  |
|  | R2_CON_ | .03 (.01) | .03 (.01) | .06 (.01) | .02 (.01) | 940 (38) | 1,028 (56) | 935 (36) | 992 (41) |
|  | R2_INC_ | .02 (.01) | .03 (.01) | .02 (.01) | .05 (.01) | 1,062 (49) | 1,121 (50) | 1,018 (41) | 1,060 (50) |
|  | New | .05 (.01) | .04 (.01) | .04 (.01) | .04 (.01) | 1,195 (80) | 1,253 (78) | 1,198 (75) | 1,206 (68) |
| Experiment 3 |  |  |  |  |  |  |  |  |  |
|  | R3 | .14 (.02) | .11 (.02) | .17 (.02) | .10 (.02) | 1,169 (67) | 1,158 (74) | 1,163 (48) | 1,218 (51) |
|  | New | .07 (.01) | .07 (.02) | .06 (.01) | .04 (.01) | 1,097 (46) | 1,141 (69) | 1,162 (48) | 1,234 (64) |

**Table S1** Mean error rates and reaction times (RTs) for recombined and new pairs split by experiment, prime load (low primed, high primed) and test task (same and reverse). Standard error of the mean within parentheses. R1 = recombined pairs in which both the selected and nonselected objects had congruent classifications; R2_CON_ = recombined pairs in which the selected object had a congruent classification and the nonselected object an incongruent classification at test; R2_INC_ = recombined pairs in which the selected object had an incongruent classification and the nonselected object a congruent classification at test; R3 = recombined pairs in which both the selected and nonselected objects had an incongruent classification at test; New = new (novel) pairs.

*Note.* Due to rounding up errors, the error rate difference between recombined and new pairs (accuracy priming) might not correspond exactly to the values shown on the figures in the main manuscript

**Analysis of RT difference**

*Experiment 1*

A 2 (prime level) × 2 (test task) mixed repeated-measures ANOVA on difference RTs revealed a significant main effect only of prime level, *F*(1, 46) = 13.65, *p* < .001, η^2^_p_ = .23, which revealed greater priming for the high than for the low primed condition. Neither the main effect of test task nor the interaction approached significance, both *F*s(1, 46) < 1.82, *p*s > .10. Indeed, when the difference between new and recombined pairs was compared between “same” and “reverse”, we did not observe any significant differences in either prime level condition, both *t*s(1, 46) < 1.74, *p*s > .09. There was, however, a substantial priming effect for both “same” and “reverse” tasks and in both prime level conditions (all *t*s > 6.02, *p*s < .001).

*Experiment 2*

A 2 (prime level) × 2 (test task) × 2 (congruence) mixed repeated-measures ANOVA on difference RTs revealed a significant main effect only of congruence, *F*(1, 46) = 45.48, *p* < .001, η^2^_p_ = .50, with larger RTs for the R2_INC_ than for the R2_CON_ pairs. None of the other main effects or interactions approached significance (all *F*s < 1.41, *p*s > .10). There was, however, a substantial priming effect for both “same” and “reverse” tasks, for both congruence types and for both prime level conditions (all *t*s > 2.83, *p*s < .01). There was no difference between R1_SAME_ and R2_CON/SAME_, *t*(94) = −1.19, *p* > .10, *d* = .42.

*Experiment 3*

A 2 (prime level) × 2 (test task) mixed repeated-measures ANOVA on difference RTs revealed a marginal effect only of prime level, *F*(1, 46) = 3.59, *p* < .06, η^2^_p_ = .07. Neither the main effect of test task or the interaction approached significance, both *F*s(1, 46) < .93, *p*s > .10. There was a trend for negative priming in the “same” task, *t*(47) = −1.61, *p* = .06, *d* = .23 (one-tailed), but not in the “reverse” task, *t*(47) = −.05, *p* > .10, *d* = .01. To test the importance of nonselected item incongruence, we compared the RT priming effect between R2_INC/SAME_ and R3_SAME_, which revealed a significant effect, *t*(94) = 4.44, *p* < .001, *d* =.91.

**Size-difficulty control analysis: Experiment 2**

In order to ensure that any RT difference between R2_CON_/R2_INC_ and intact/new pairs were not due to different levels of difficulty in categorizing R2_CON_ or R2_INC_ pairs, 12 participants, who did not take part in any of the experiments reported in this study, were asked to perform the “Which is bigger?” task on intact, R2_CON_, R2_INC_, and new pairs randomly intermixed. A one-way repeated-measures ANOVA using pair rype (intact, R2_CON_ and R2_INC_, new) as a factor, was conducted. The results showed that neither accuracy, *F*(3, 33) = 1.89, *p* > .10, η^2^_p_ = .15, nor RTs, *F*(3, 33) = .16, *p* > .10, η^2^_p_ = .01, differed among conditions, indicating that all conditions were matched in terms of size-judgement difficulty.

##### Size-difficulty control analysis: Experiment 3

We asked 10 participants, who did not take part in any of the experiments reported in the present study, to perform the “Which is bigger?” task on intact, R, R3, and new pairs in order to examine for potential RT differences due to the difficulty arising from R3 pairs. A repeated-measures ANOVA was conducted. The ANOVA revealed a significant effect of type of association on both accuracy, *F*(3, 27) = 5.63, *p* < .01, η^2^_p_ = .39, and RTs, *F*(3, 27) = 16.85, *p* < .001, η^2^_p_ = .65. Post hoc comparisons revealed that only R pairs were significantly judged more accurately and faster than any other type of association. This was expected because the two objects in R pairs were separated by three size steps (A–D associations; see Method section), whereas for R3 pairs the objects were separated by only one (B–C associations; see Method section). Because R pairs were only used to make the construction of R3 pairs possible, they were not included in further statistical analysis.

**Replication of the outcome congruence effect: Experiment 3**

In order to test the outcome congruence effect, we contrasted recombinations in which decision/action and classification responses were congruent for both the selected and nonselected items (R1) with recombinations in which the three response levels were incongruent for both recombined items (R3_SAME_). As expected, this difference was also highly significant, *t*(94) = 8.35, *p* < .001, *d* = 1.66.

**Analysis of incorrect responses RTs:- Experiments 2 and 3**

We also analyzed the RTs for incorrect responses. The comparison between R2_INC/REV_ and R2_INC/SAME_ was significant, *t*(7) = 2.25, *p* < .05, *d* = .79 (one-tailed). As predicted, we also observed faster RTs for incorrect responses to R2_CON/SAME_ relative to R2_CON/REV_, but this test did not reach significance, *t*(5) = −.95, *p* > .10, *d* = .39, although this is likely to be related to the small number of observations. Likewise, inaccurate responses to R3_SAME_ (which is associated with inappropriate response activation for the nonselected item) were faster than inaccurate responses to R3_REV_ (which is associated with appropriate inhibition for the nonselected item)*, t*(30) = −2.95, *p* < .01, *d* = .53 (see Fig. S1).


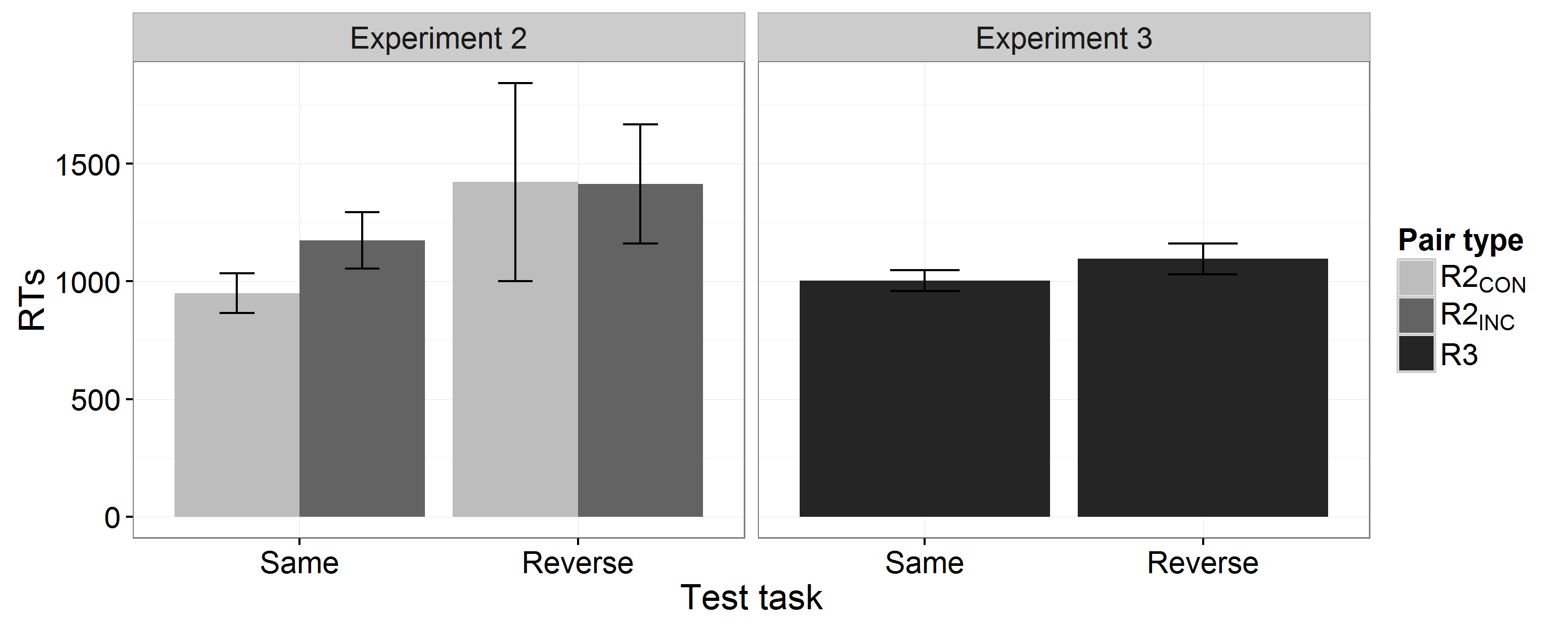


**Fig. S1** Reaction times (RTs) for incorrect responses for recombinations in Experiment 2 (left panel) and Experiment 3 (right panel) during the “same” and “reverse” tasks. Error bars represent the standard error of the mean
